# A. V. M. A.

**Published:** 1899-12

**Authors:** 


### A. V. M. A.

### LIST OF APPOINTMENTS FOR 1899–1900.

*Executive Committee*—Tait Butler, Chairman, Indianapolis, Ind.; D. E. Salmon, Washington, D. C.; W. Horace Hoskins, Philadelphia, Pa.; M. H. Reynolds, St. Anthony's Park, Minn.; A. W. Clement, Baltimore, Md.; A. T. Peters, Lincoln, Neb.; R. R. Bell, New York City, N. Y.

*Finance*—Lemuel Pope, Jr., Chairman, Portsmouth, N. H.; M. Stalker, Ames, Iowa; G. A. Johnson, Sioux City, Iowa.

*Publication*—M. H. Reynolds, Chairman, St Anthony's Park, Minn.; R. P. Lyman, Hartford, Conn.; John J. Repp, Ames, Iowa; R. R. Bell, New York City, N. Y.; S. Stewart, Kansas City, Kansas.

*Intelligence and Education*—W. H. Dalrymple, Chairman, Baton Rouge, La.; James Law, Ithaca, N. Y.; John W. Adams, Philadelphia, Pa.; N. S. Mayo, Storrs, Conn.; A. H. Baker, Chicago, Ill.

*Diseases*—C. A. Cary, Chairman, Auburn, Alabama; James B. Paige, Amherst, Mass.; John P. Turner, Washington, D. C.; E. M. Nighbert, Mt. Sterling, Ill.; M. E. Knowles, Helena, Montana.

*Army Legislation*—D. E. Salmon, Chairman, Washington, D. C.; R. S. Huidekoper, Washington, D. C.; W. Horace Hoskins, Philadelphia, Pa.; M. Stalker, Ames, Iowa; A. W. Clement, Baltimore, Md.

*Resolutions*—L. H. Howard, Chairman, Boston, Mass.; Wm. Herbert Lowe, Paterson, N. J.; C. E. Cotton, Minneapolis, Minn.; L. A. Merillat, Chicago, Ill.; W. B. Niles, Ames, Iowa.

*Resident State Secretaries*—Alabama, R. H. Drummond, Birmingham. Arizona, J. C. Norton, Phœnix. California, Fred. E. Pierce, 1724 Webster Street, Oakland. Colorado, Charles Gresswell, Montclair. Connecticut, R. P. Lyman, 997 Main Street, Hartford. Cuba, C. D. McMurdo, Santiago. Delaware, H. P. Eves, 507 W. Ninth Street, Wilmington. District of Columbia, A. M. Farrington, 1436 Chapin Street, Washington. Georgia, Ray J. Stanclift, Americus. Hawaiian Islands, W. T. Monsarrat, Honolulu. Illinois, E. M. Nighbert, Sterling. Indiana, J. R. Mitchell, Evansville. Iowa, T. A. Bown, Chariton. Kansas, W. H. Richards, Emporia. Kentucky, J. W. Jamieson, Paris. Louisiana, W. H. Dalrymple,

Baton Rouge. Manitoba, W. J. Hinman, Winnipeg. Maryland, E. C. Fox, 2823 Huntingdon Street, Baltimore. Massachusetts, Benj. D. Pierce, 27 Stanford Street, Springfield. Michigan, George W. Dunphy, Quincy. Minnesota, S. A. Ward, St. Cloud. Mississippi, J. C. Roberts, Agricultural College. Missouri, Charles Ellis, 3230 Locust Street, St. Louis. Montana, M. E. Knowles, Helena. Nebraska, V. Schaefer, Tekamah. New Hampshire, Lemuel Pope, Jr., 101 State Street, Portsmouth. New Jersey, J. Payne Lowe, 19 Bloomfield Avenue, Passaic. New York, Wm. Henry Kelly, 195 Western Avenue, Albany. North Carolina, A. S. Wheeler, Biltmore. North Dakota, T. D. Hinebauch, Tower City. Nova Scotia, Wm. Jakeman, Halifax. Ohio, T. Bent Cotton, Mt. Vernon. Ontario and Quebec, Charles H. Higgins, 168 Mansfield Street, Montreal. Oregon, Wm. McLean, 328 Fourth Street, Portland. Pennsylvania, W. H. Ridge, Trevose. Puerto Rico, G. E. Griffin, Mayaguez. Rhode Island, J. M. Armstrong, Providence. South Carolina, Benj. McInnes, Charleston. Tennessee, Joseph Plaskett, 529 Broad Street, Nashville. Texas, M. Francis, College Station. Virginia, E. P. Nies, Blacksburg. Washington, S. B. Nelson, Pullman. Wisconsin, R. H. Harrison, 83 Fourteenth Street, Milwaukee.

